# GLUT 5 Is Not Over-Expressed in Breast Cancer Cells and Patient Breast Cancer Tissues

**DOI:** 10.1371/journal.pone.0026902

**Published:** 2011-11-02

**Authors:** Gayatri Gowrishankar, Sabine Zitzmann-Kolbe, Anitha Junutula, Robert Reeves, Jelena Levi, Ananth Srinivasan, Kjerstin Bruus-Jensen, John Cyr, Ludger Dinkelborg, Sanjiv S. Gambhir

**Affiliations:** 1 Molecular Imaging Program, Department of Radiology, Division of Nuclear Medicine, Stanford, California, United States of America; 2 Bayer Schering Pharma AG, Global Drug Discovery, Bayer Schering Pharma AG, Berlin, Germany; 3 Department of Bioengineering and Materials Sciences & Engineering, Stanford, California, United States of America; National Institute of Health, United States of America

## Abstract

F18 2-Fluoro 2-deoxyglucose (FDG) has been the gold standard in positron emission tomography (PET) oncologic imaging since its introduction into the clinics several years ago. Seeking to complement FDG in the diagnosis of breast cancer using radio labeled fructose based analogs, we investigated the expression of the chief fructose transporter-GLUT 5 in breast cancer cells and human tissues. Our results indicate that GLUT 5 is not over-expressed in breast cancer tissues as assessed by an extensive immunohistochemistry study. RT-PCR studies showed that the GLUT 5 mRNA was present at minimal amounts in breast cancer cell lines. Further knocking down the expression of GLUT 5 in breast cancer cells using RNA interference did not affect the fructose uptake in these cell lines. Taken together these results are consistent with GLUT 5 not being essential for fructose uptake in breast cancer cells and tissues.

## Introduction

Breast cancer is the second leading cause of cancer deaths in women today and is the most common form of cancer among women excluding non-melanoma skin cancers. The World Health Organization estimated that worldwide, in excess of 1.2 million people were diagnosed with breast cancer in 2005 [1]. Breast cancer cells have a high level of glucose uptake and metabolism, a common characteristic of most cancer cells. This increased glucose uptake and aerobic metabolism forms the basis for assessment of tumor metabolism and response to therapy by positron emission tomography (PET) imaging with FDG (F-18 2-fluoro-2′-deoxyglucose) [2], [3], [4]. Due to poor imaging of noninvasive breast cancer [5], ^18^FDG-PET has been mostly performed on invasive breast cancer. The success of ^18^FDG-PET for the initial detection and diagnosis of primary breast cancer varies greatly [6], which is mainly attributed to different metabolism levels in different types of tumors, e.g. infiltrating ductal carcinoma has a higher level of ^18^FDG uptake than infiltrating lobular breast cancer. Also, the staging of axillary lymph nodes with ^18^FDG-PET has produced mixed results. Patients with invasive breast cancer routinely undergo lymphoscintigraphy and axillary lymph node dissection (ALND). Sentinel node biopsies (SNB) are also performed. Comparison of these methods with ^18^FDG-PET shows very different sensitivities and specificities for ^18^FDG-PET, usually (but not always) with higher sensitivity and lower specificity, and many recent studies suggest that ^18^FDG-PET may not have a sufficiently high negative predictive value to justify forgoing ALND. One reason is the difference in minimal size of the lesion that can be detected. Even with modern clinical PET systems the limit of spatial resolution is approximately 6.0 mm, which is consistent with the results that ^18^FDG activity was very low or non-detectable in patients with tumors sizes ranging from 0.4 to 1.5 cm.

Glucose, galactose and fructose serve as basic fuel molecules for many cells, and these molecules require transporters for entry and exit from those cells. Three distinct groups of hexose transporters have been identified and classified based on their dependence on cellular energy [7], [8]. The first class of transporters, GLUT1-4 is primarily glucose transporters with distinct tissue distributions. The presence of the GLUT1 transporter is important for the imaging of tumor and inflammatory tissues with FDG because FDG is transported mainly by GLUT1. The second class of transporters is exemplified by GLUT5, GLUT7, GLUT9 and GLUT11 all of which are previously known fructose transporters. Uptake of fructose is thought to be mediated mainly through GLUT5, and based on similarity of the different isoforms within this class; GLUT7, GLUT9 and GLUT11 are also fructose transporters. GLUT5 does not transport glucose and is expressed mainly in small intestine, sperm cells and brain with very low level expression in muscle and adipose tissue. GLUT5 transports fructose with high affinity. At normally low physiological concentrations of fructose *in vivo*, GLUT5 likely mediates >90% of fructose uptake owing to the order of magnitude difference in respective Km values for GLUT5 and the other fructose transporters. The remainder of the transporters represents the third class of GLUT transporters, which are largely as yet uncharacterized with limited knowledge regarding exact cellular functions.

GLUT transporters are intrinsic membrane proteins differing in tissue specific expression and response to metabolic and hormonal regulation [9]. Although a number of different isoforms of GLUT have been identified, they all appear to share a common membrane topology possessing a large highly conserved transmembrane domain, with less conserved assymetric-membrane cytoplasmic and exoplasmic domains [10]. The transmembrane domain is composed of twelve membrane-spanning helices containing a water filled pathway through which the substrate moves [11], [12]. The cytoplasmic domain contains a short N-terminal segment, a large cytosolic loop and a large C-terminal segment. The exoplasmic domain consists of a large loop structure bearing a single N-linked oligosaccharide moiety. Isoform specific amino acid sequences are found in the cytoplasmic and exoplasmic domains indicating that they are responsible for tissue-specific regulation of transporter function. The primary structure of the transmembrane domain is largely conserved, suggesting that the glucose channel is essentially identical in structure among the different isoforms.

Localization, expression and regulation of the GLUT family members are tissue and cell-specific. As new GLUT isoforms are continually being discovered and characterized in various cell types, their involvement in disease states is under constant review. In cancer cells, which have broken free from the normally tight biochemical regulation, aberrant expression of the GLUT family members is often observed, presumably to help provide the energy required for further uncontrolled proliferation and metastasis.

It has been well established that the GLUT1 transporter is highly expressed in breast cancer cells compared to normal counterparts [13]. Vera and co-workers [14] have analyzed expression and function of glucose transporters in breast cell lines (MCF-7 and MDA-MB-468) and in normal and neoplastic human breast tissue. According to their results in addition to GLUT1, human breast tissue selectively expresses the high affinity fructose transporter GLUT5. Another report by the same group further substantiated this claim by performing an extensive immunohistochemistry study in 33 breast tissues samples (representing invasive ductal carcinoma) [15]. Knocking down GLUT5 by antisense RNA seemed to inhibit the proliferation of MCF7 and MDA-MB-231 cells [16]. It has also been demonstrated that several different cancer cell lines, including breast cancer, melanoma, Caco-2 (colonic cancer) and leukemia over-express active GLUT5 transporters (15).

While GLUT1 is over-expressed in breast cancer and present in normal breast, as per the references cited above GLUT5 is largely absent in normal tissue with high expression levels in human breast cancer tissue. Hence, specific substrates that are either transported by GLUT5 or retained and/or inhibitors of fructose transport with high affinity for GLUT5 should be more effective imaging agents than those targeting GLUT1.

In this report we have tried to validate the expression of GLUT 5 in the breast cancer cell lines-MCF7 and MDA MB 468 (previously reported to express GLUT 5) and in patient breast cancer tissues with the intention of exploring the possibility of using radiolabeled fructose or fructose analogs as PET tracers. Our results indicate that there is no over-expression of GLUT5 in breast cancer cells and patient breast cancer tissues as previously reported. The possible reasons for the discrepancy in observations are discussed and some crucial experiments that would be essential to understand the utility of fructose based tracers for breast cancer imaging are also mentioned in the discussion.

## Materials and Methods

### Cell culture and siRNA transfections

MDA-MB-468 and MCF 7 cells were grown as per ATCC (American Type Culture Collections) conditions in Leibovitz L15 and RPMI 1640 respectively. The former needs to be cultured in the absence of CO_2_. Stealth siRNA (short-interfering RNA) against GLUT 5 (accession no: NM_003039) were ordered from Invitrogen. Three different siRNA against GLUT 5 were obtained and tested. Negative control siRNA was also ordered from Invitrogen. RNAimax (transfection reagent) from Invitrogen was used to reverse- transfect the siRNA into MCF7 and MDA-MB-468 cells. In brief, 10 nM of the GLUT 5 specific siRNA and 40 nM of the negative control were mixed with the appropriate amount of Lipofectamine RNAimax in Optimem (serum free media, Invitrogen) and added to the wells (either 24 well or 6 well dishes as needed), where they were allowed to incubate for 20 mins at room temperature. The cells were then counted, diluted (50,000 cells/500 µl) in antibiotic free media and added to the wells containing the siRNA-lipofectamine mixture. The cells were incubated for 48 hrs before assaying for knockdown.

### Cell uptake studies

2×10^5^ cells (per well) were plated in a 24-well plate 24 hrs before the assay in normal growth media. After removing the media, 1 µCi of either C14-fructose (97% pure, 250–360 mCi/mmol) or C14- glucose(98% pure, 50–60 mCi/mmol) (Moravek Chemicals, La Brea, California) was added to each well in HBSS (Hank's buffered saline solution or PBS (Phosphate buffered saline) buffers as required by the assay, and incubated for the specified time points in a 37°C incubator. The HBSS buffer has 5 mM glucose (physiological levels) in addition to salts. After the incubation, cells were washed twice with cold PBS and 500 µl of 0.1N Sodium Hydroxide was added to the wells to lyse the cells. 10 ml of the liquid scintillation cocktail was added to 250 µl of the lysate and the vials placed in a liquid scintillation counter for determining the cell associated radioactivity. 50 µl of the lysate was used for determining the protein concentration using Bradford assay.

### RNA extraction and analysis

Total RNA was extracted from the cells using the Qiagen RNeasy kit following the manufacturer's instructions. An aliquot of the extracted RNA was subjected to DNase I treatment (Invitrogen), and the DNase I treated RNA was used to synthesize cDNA. This cDNA was then used for both qualitative RT-PCR (using GoTaq PCR master mix, Promega) and quantitative real-time PCR (Qiagen Sybr Green mix). The primers used were as follows:

GLUT 5 FW: tcgtgcctgcgatcttaatg

GLUT 5 BW: cagccatctacgtttgcaag

Actin FW: ggtcatcttctcgcggttggccttgg

Actin BW: ccccaggcaccagggcgtgat

### Immunohistochemistry

The study was performed by LifeSpan (Seattle, WA) and used full slides of normal breast (n = 10), mastitis (n = 10), infiltrating lobular carcinoma with benign tissue (n = 10) and a breast carcinoma tissue microarray (n = 40, LS-SBRCA1). For screening normal tissues a tissue microarray from LifeSpan was used (LS-SNL2), containing duplicate specimens each of normal human adrenal, bladder, brain cortex, breast, colon, heart, kidney cortex, kidney medulla, liver, lung, ovary, pancreas, placenta, prostate, skeletal muscle, skin, small intestine, spleen, stomach, testis, thymus, thyroid, tonsil, and uterus. Antibody GLUT 5 (Abcam, catalog #AB36057 demonstrated the highest signal-to-noise ratio at a concentration of 10 ug/ml on formalin-fixed, paraffin embedded tissues. Antibody GLUT 5 was used as the primary antibody, and the principal detection system consisted of a Vector anti-rabbit secondary (BA-1000) and a Vector ABC-AP kit (AK-5000) with a Vector Red substrate kit (SK-5100), which was used to produce a fuchsia-colored deposit. Tissues were also stained with positive control antibodies (CD31 and vimentin) to ensure that the tissue antigens were preserved and accessible for immunohistochemical analysis. The negative control consisted of performing the entire immunohistochemistry procedure on adjacent sections in the absence of primary antibody. Slides were imaged with a DVC 1310C digital camera coupled to a Nikon microscope.

### Statistical analysis

Online statistical analysis tools from GraphPad software were used for our statistical analysis. An unpaired t-test using mean, SEM (standard error of mean) and N (number) was done to calculate a two-tailed P value.

### Ethics Statement

The immunohistochemistry study carried out at LifeSpan is done on patient tissue samples that are provided to LifeSpan by a source unknown to us. LifeSpan obtains human samples from a variety of third party sources including hospital pathology departments and organ donor organizations (not directly organ donors). Each source complies with IRB approved protocols and patient consent release policies appropriate for that source. All tissues acquired by Lifespan have been cleansed of patient identifiers prior to receipt by LifeSpan to preserve confidentiality.

## Results

### GLUT 5 expression in breast cancer cell lines

**Figure 1:**
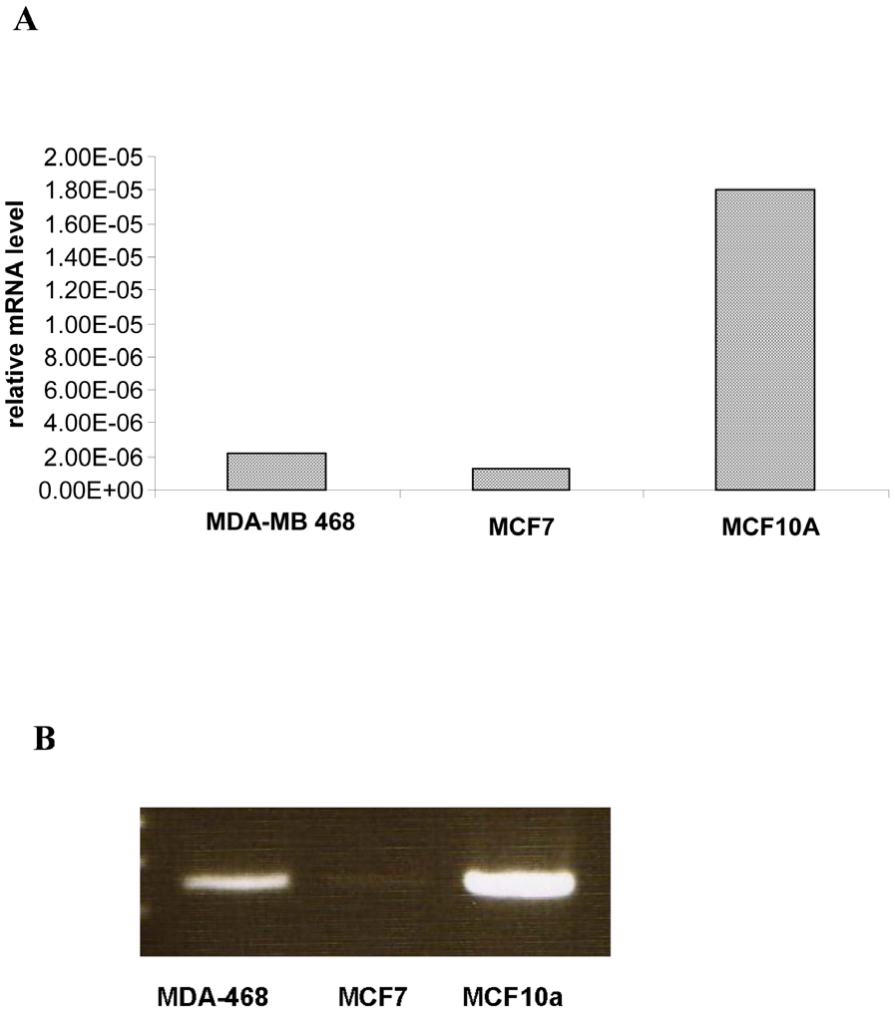
GLUT 5 mRNA levels by quantitative real time PCR. RNA from MCF7, MDA MB 468 and MCF10A were extracted, converted to cDNA and quantified by real time PCR (fig 1A) and the products also visualized on a 1% agarose gel (fig 1B).

GLUT 5 expression in the MCF7, MDA-MB4-68 and MCF10A cell lines was evaluated at the mRNA level by quantitative real-time PCR. The latter-MCF10A is an immortalized breast epithelial cell line often used by researchers as ‘normal’ control for breast cells. As observed in Fig 1A, the MCF 10A cell line had the highest levels of GLUT 5 mRNA. Both MCF7 and MDA-MB-468 cell lines had low levels of GLUT5 mRNA. A representative gel picture is shown in Fig 1B.

### Fructose uptake in breast cancer cell lines

**Figure 2:**
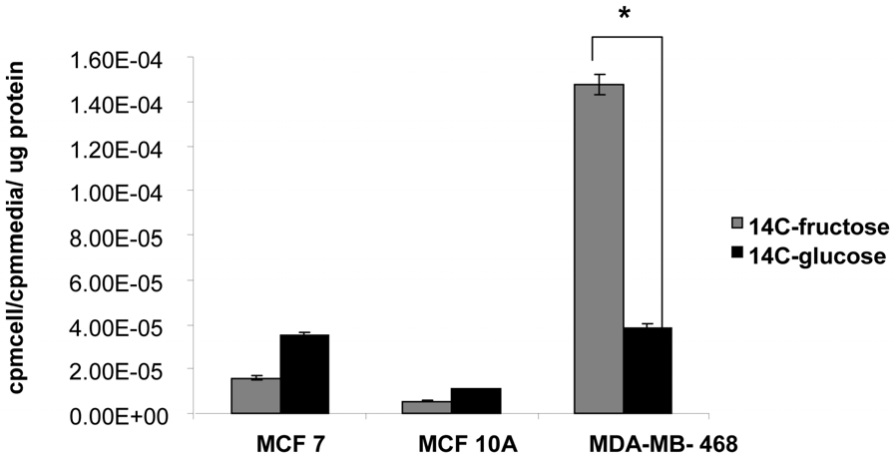
Glucose and Fructose are both taken up by breast cancer cell lines. MCF 7 and MDA MB 468 (breast cancer cell lines) and MCF10A (breast epithelial cell line) were incubated with 1 µCi each of C14 glucose and C14 fructose for 10 mins in HBSS buffer. The activity associated with the cells was counted and normalized to activity in the media and to total protein mass associated with the cells. * Indicates p<0.0001 as determined by an unpaired student t-test described in methods. The error bars represent S.E.M of triplicates.

MCF7, MDA-MB-468 and MCF10A cells were incubated with C14 glucose and C14 fructose for 10 mins, to examine the relative uptake of both tracers. The uptake was done in HBSS buffer which has physiological levels of glucose (5 mM). Under these conditions, the cancer cell lines-MCF7 and MDA-MB-468 take up more of the sugars as compared to the normal MCF10A cell line (Fig 2). The uptake of both sugars in MCF7 and MCF10A is comparable, but the MDA-MB-468 takes up significantly more of the C14 fructose as compared to the C14 glucose (p<0.0001). This result is in contrast to the mRNA expression data shown in Fig 1, where the MCF10A cell line had the highest levels of the GLUT5 mRNA. The above two experiments gave us the first hint that there was no correlation between the level of GLUT 5 mRNA and the fructose uptake across the breast cancer cell lines that we tested.

### siRNA knockdown of GLUT 5 in breast cancer cell line

**Figure 3:**
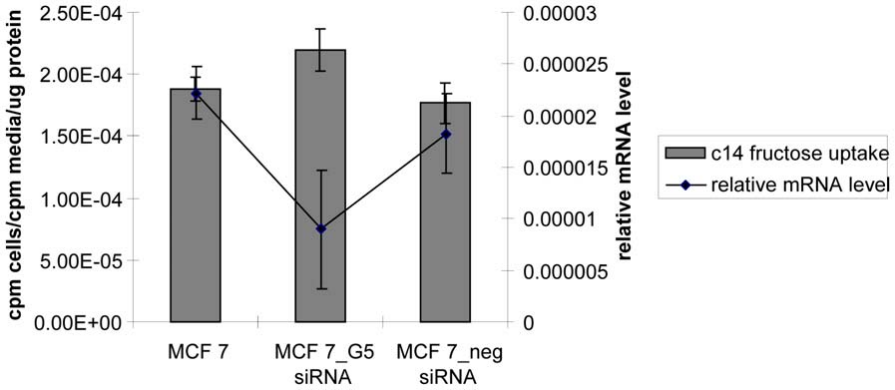
Knocking down GLUT 5 mRNA in MCF7 cells has no effect on fructose uptake. GLUT 5 specific siRNA and negative control siRNA were transfected into MCF7 cells. 48 hrs after transfection cells were simultaneously interrogated for both mRNA levels (using quantitative real time PCR) (right Y axis) and for fructose uptake (left Y axis) by incubating with C14 labeled fructose. The error bars represent S.E.M of triplicates.

siRNA were designed against the GLUT 5 mRNA. The GLUT 5 specific siRNA and a non-specific negative control siRNA were transfected into MCF7 cells. 48 hrs after transfection, lysates were prepared for RNA analysis. Quantitative RT-PCR showed that the GLUT 5 mRNA levels had been knocked down by 59% (Fig 3). In parallel fructose uptake was evaluated at 48 hrs. post-transfection in the transfected and non-transfected cells. As shown in Fig 3, there was no significant change in the fructose uptake (p = 0.46) although the GLUT 5 mRNA had been knocked down. These experiments thus indicate that GLUT 5 may not be essential for fructose transport into these breast cancer cell lines contrary to what has previously been reported by Vera et al [14].

### Cytochalasin B treatment blocks fructose transport into MDA-MB468 cells

**Figure 4:**
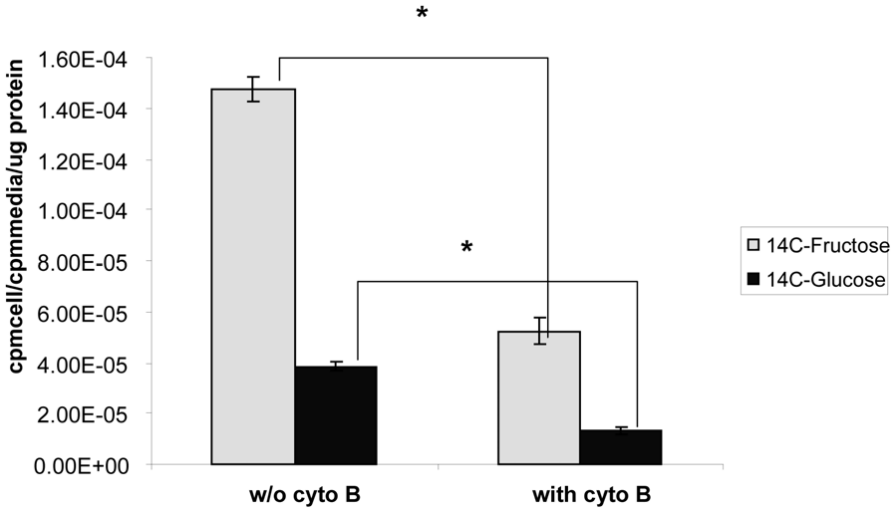
Cytochalasin B treatment affects fructose uptake in MDA MB 468 cells. MDA MB 468 cells were exposed to 100 µM cytochalasin B for 30 mins. Then 1 µCi of C14 fructose or glucose was added to the cells, and the uptake was continued for 10 mins in HBSS. The activity associated with the cells was then counted and normalized to activity in the medium and to total protein mass. * indicates p values <0.05. The error bars represent S.E.M of triplicates.

The alkaloid Cytochalasin B is a known inhibitor of hexose transport via GLUT 1, 2, 3 and 4 through its actions on the cytoskeleton. However fructose transport through GLUT 5 is insensitive to cytochalasin B treatment [17]. MDA-MB-468 cells were treated with 100 uM of cytochalasin B and the uptake of C14 fructose and C14 glucose was evaluated in the presence/absence of cytochalasin B (Fig 4). The glucose uptake was blocked by about 34% (p =  0.0004) and the fructose uptake was blocked by about 35% (p = 0.0002). Since the fructose uptake was sensitive to cytochalasin B treatment, this indicates that fructose uptake in MDA-MB-468 cells does not rely solely on GLUT5.

### Immunohistochemistry

We wanted to evaluate GLUT5 expression in normal vs. breast cancer tissue samples. The study evaluated GLUT5 expression in full slides in normal breast (n = 10), mastitis (n = 10), infiltrating lobular carcinoma with benign tissue (n = 10) and GLUT5 expression in a breast carcinoma tissue microarray (n = 40).

**Figure 5:**
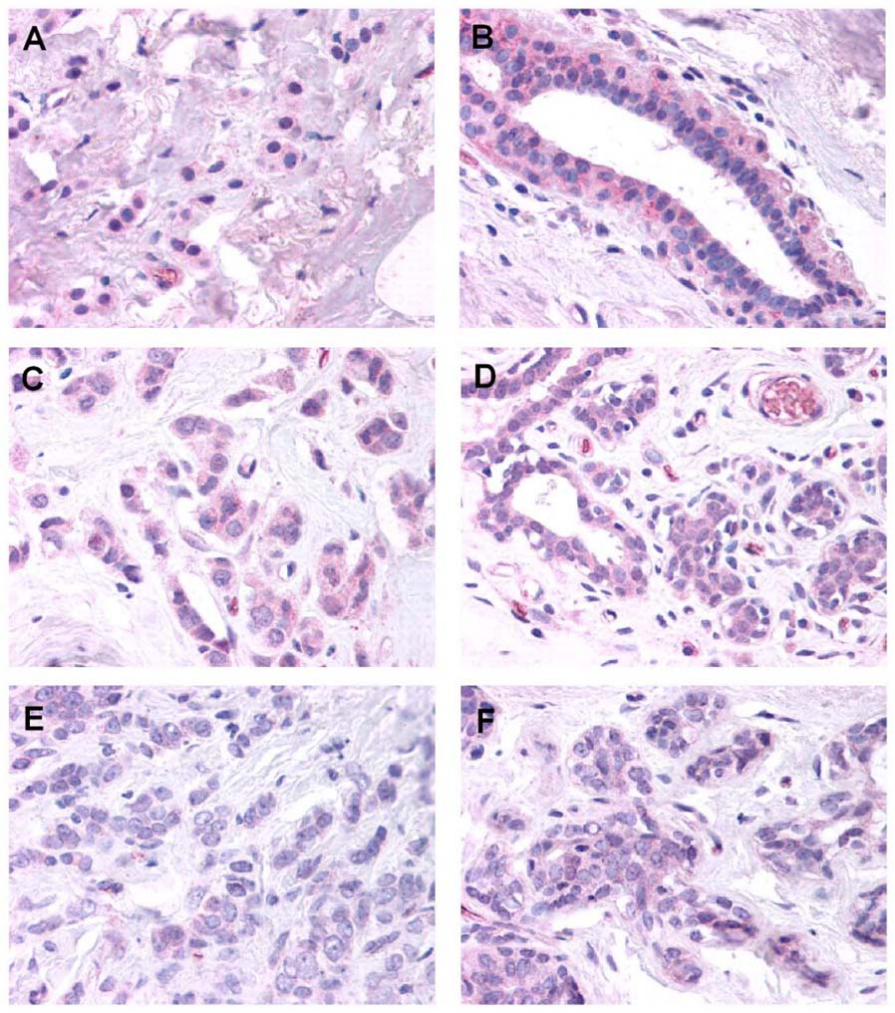
Immunohistochemistry study of breast tissue. Immunohistochemical staining of breast carcinoma and adjacent normal breast tissue with Glut5-antibody. **A**, **C** and **E** are infiltrating lobular carcinoma and **B**, **D** and **F** the corresponding normal breast tissue. (Magnification is 40x).

In breast tissue samples of benign breast, glandular epithelium showed positive cytoplasmic and slightly weaker nuclear staining. Occasionally, mast cells and plasma cells were also positive. In samples of breast with mastitis (data not shown), glandular epithelium and the surrounding myoepithelial cells showed slightly less intense staining in most samples compared to normal samples. In the inflammatory infiltrate, focal positive staining was present in plasma cells, and weaker staining was present in macrophages and giant cells, but most neutrophils were negative. In breast tissue samples containing infiltrating lobular carcinoma with surrounding benign breast tissue (Fig 5), most samples showed weak staining in malignant cells and endothelium. Staining often appeared to be slightly more intense in benign glands compared to malignant cells. There was no differential staining in vessels, inflammatory cells, stroma, or entrapped benign epithelium in areas of tumor compared to adjacent benign areas. In a panel of breast carcinomas consisting of small cores of tissue taken from regions of tumor, most samples showed weak staining in malignant cells (see Table 1).

**Table 1:** Staining intensities from 40 breast cancer samples stained with GLUT 5-antibody.

**Staining Intensity**	**0**	**1**	**1**–**2**	**2**	**2**–**3**	**3**	**4**
**Number of Samples**	4	12	12	9	2	1	0

**Figure 6:**
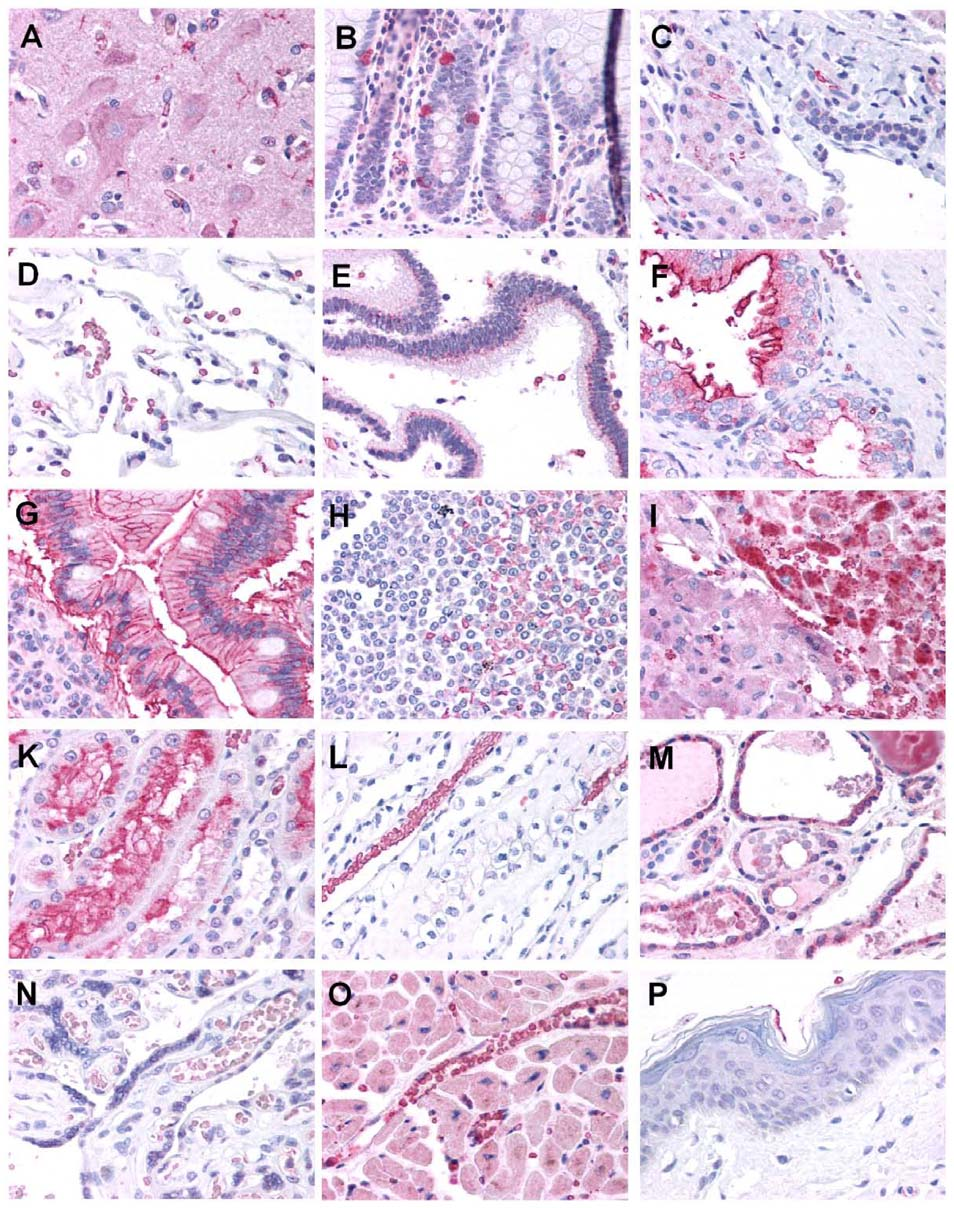
Immunohistochemistry study of a tissues array. Immunohistochemical staining of tissue microarray with Glut5-antibody. **A**: Brain cortex (neurons and microglia) **B**: Colon mucosa with intraepithelial neuroendocrine cells; **C**: Liver (hepatocytes and bile duct); **D**: Lung (alveoli and erythrocytes); **E**: Pancreas (duct); **F**: Prostate (glands and stroma); **G**: Small intestine (villi); **H**: Spleen (white pulp and red pulp); **I**: Adrenal (junction of cortex and medulla); **K**: Kidney cortex (renal tubular epithelium); **L**: Kidney medulla (medulla and erythrocytes); **M**: Thyroid (follicular epithelium); **N**: Placenta (villi); **O**: Heart (cardiac myocytes and capillaries); **P**: Skin (Squamous Epithelium) (Magnification is 40x).

A normal tissue microarray (n = 2 per tissue) was also examined for GLUT5 expression (Fig 6). In the normal tissues, the most significant staining was identified in renal proximal convoluted tubules and erythrocytes. Prominent staining was also present in occasional macrophages, lymphocytes, gastrointestinal neuroendocrine cells, the adrenal cortex, and microglia in the brain. Occasionally, positive membranous and perinuclear staining was present in intestinal epithelium and pancreatic ducts. Less intense or focal staining was also identified in cardiomyocytes, breast epithelium, colonic glandular epithelium, brain neurons, hepatocytes, ovarian follicles, and thyroid follicular epithelium. Staining was particularly weak or absent in skin, pancreatic acini and islets, and placental villi, which helps to confirm the specificity of this antibody and distinguish this target from GLUT1, which would be expected to be positive in these tissues.

## Discussion

In this report we tried to validate the expression of the fructose transporter-GLUT5 in breast cancer cells and patient tissues. Our work was based on the observations of Zamore-Leon et. al. who previously reported specific expression of the GLUT5 in primary breast cancer tissues (n = 20) with no staining observed in normal mammary tissue [14]. Interestingly an erratum was published as a follow up later that year that said their statement ‘no staining in normal mammary tissue’ was not correct and that they did observe some staining in normal tissue [18]. A second publication by Godoy et. al. [15] also detected GLUT 5 in human breast ductal carcinoma (n = 33). However they also reported weak to moderate staining in normal breast tissue. An important observation though was that in contrast to GLUT1 which localized to the plasma membrane, all of the samples which were positive for GLUT5 showed cytoplasmic staining. This may indicate that the GLUT5 may need to be activated to localize to the plasma membrane. Two other publications that investigated GLUT5 expression by immunohistochemistry [13], [19] reported weak to no staining in neoplastic breast tissues. Our own results do not support the observation by Zamore-Leon et. al. (Fig 5 and 6). We did not observe any significant over-expression of GLUT5 in breast cancer tissue. While one could attribute the differences to the use of different antibodies, in our study (conducted in collaboration with Lifespan Inc.) we tested two different antibodies in phase I after which one was chosen for phase II IHC staining of the actual breast tissues. Interestingly we found inter-sample variability with some tumors showing higher expression and other tumors showing lower expression, but within a sample containing both normal breast and breast tumor tissue GLUT5 expression showed no significant differences between normal breast and breast tumor tissue. We also observed moderate staining in immune cells which would argue against the presumption that fructose may be preferentially taken up by breast cancer cells. The human protein atlas (www.proteinatlas.org) which is a free online database of IHC studies available to researchers, stated that “most malignancies displayed negative or weak immunoreactivity in some cells” for GLUT5 [20]. In further support of our IHC results, an examination of GLUT 5 mRNA levels by microarray analysis did not show a significant difference between breast cancer tissue and normal breast tissues (data not shown).

While our immunohistochemistry data shows no evidence to indicate that GLUT5 is greatly over-expressed in breast cancer tissues, we do see fructose uptake in the breast cancer cells lines-MCF7 and MDA-MB 468, in contrast to the immortalized breast epithelial cell line-MCF10A (see Fig 2). This could indicate that fructose itself could be a marker that distinguishes breast cancer cells from normal cells. A recent report by Sreekumar et al showed that fructose was one of the metabolites that came up in a large scale metabolomics study of prostate cancer tissue differentiating benign from PCAs [21]. Moreover work from our own lab has shown that fluorescently labeled fructose derivatives could be taken up by breast cancer cells [22].

This uptake of fructose in the breast cancer cell lines-MCF7 and MDA MB 468- was sensitive to cytochalasin B (Fig 4) –a potent inhibitor of glucose uptake by GLUT1[17]. Fructose uptake by GLUT5 on the other hand should not be affected by cytochalasin B [17], [23] but it was. Further knocking down GLUT5 expression by RNA interference also did not affect fructose uptake (Fig 3). The fructose uptake in the cell lines also did not correlate to GLUT5 mRNA levels (Fig 1 and 2). All these experiments are consistent with the possibility that fructose uptake in the breast cancer cell lines MCF7 and MDA-468 is not solely dependant on GLUT5 expression. A limitation of the current study is the lack of western blotting data showing protein expression or lack of in the MCF 7 and MDA MB 468 cells. We did attempt western blotting with different GLUT5 antibodies but were unable to see a clear band of the right size in MDA-MB 468 cells (data not shown). We did however see some bands in the MCF 7 cells. Since our focus was to evaluate GLUT5 expression in tissue sections by IHC we did not further pursue the western blotting.

In this study we did not study the expression of other fructose transporters (GLUT 2, GLUT 7 and GLUT 11) in breast cancer tissues or cell lines. It may very well be that fructose could still be a substrate of interest to the imaging community as was observed in two recent reports [24], [25] and that increased fructose uptake in breast cancer cells is mediated by one of the other fructose transporters. Future studies would need to examine if indeed fructose is taken up better than glucose *in vivo* in breast cancer animal models through a biodistribution study using C14 labeled fructose.

### Conclusion

In conclusion, we find no evidence for GLUT 5 over-expression in breast cancer cells and tissues contrary to previous reports by others. More studies are needed to determine if radiolabeled fructose/fructose analogues could be used as metabolic PET tracers in the imaging of breast.
